# Prediction of lesion-based response to PRRT using baseline somatostatin receptor PET

**DOI:** 10.3389/fmed.2025.1523862

**Published:** 2025-03-14

**Authors:** Anas Aouf, Tilman Speicher, Arne Blickle, Moritz B. Bastian, Caroline Burgard, Florian Rosar, Samer Ezziddin, Amir Sabet

**Affiliations:** ^1^Department of Nuclear Medicine, University Hospital Bonn, Bonn, Germany; ^2^Department of Nuclear Medicine, Saarland University Hospital, Homburg, Germany

**Keywords:** neuroendocrine tumors, response prediction, peptide receptor radionuclide therapy, [^177^Lu]Lu-octreotate, [^68^Ga]Ga-DOTATOC-PET/CT

## Abstract

**Aim:**

The heterogeneous expression of somatostatin receptors in gastroenteropancreatic neuroendocrine tumors (GEP-NET) leads to significant intra-individual variability in tracer uptake during pre-therapeutic [^68^Ga]Ga-DOTATOC PET/CT for patients receiving peptide receptor radionuclide therapy (PRRT). This study aims to evaluate the lesion-based relationship between receptor-mediated tracer uptake and the functional response to PRRT.

**Methods:**

A retrospective analysis was conducted on 32 patients with metastatic GEP-NET (12 pancreatic and 20 non-pancreatic), all treated with [^177^Lu]Lu-octreotate (4 cycles, with a mean of 7.9 GBq per cycle). [^68^Ga]Ga-DOTATOC PET/CT was performed at baseline and 3 months after the final PRRT cycle. Tumor uptake was quantified using the standardized uptake value (SUV). For each patient, 2 to 3 well-delineated tumor lesions were selected as target lesions. SUV_max_, SUV_mean_ (automated segmentation with a 50% SUV_max_ threshold), and corresponding tumor-to-liver ratios (SUV_maxT/L_ and SUV_meanT/L_) were calculated. Functional tumor response was assessed based on the relative change in metabolic tumor volume (%ΔTV_PET_). The correlation between baseline SUV parameters and lesion-based functional response was analyzed using Spearman’s rank correlation.

**Results:**

A total of 71 lesions were included in the analysis. The mean baseline SUV_max_ and SUV_mean_ were 28.1 ± 15.9 and 13.6 ± 5.1, respectively. Three months after PRRT completion, the mean %ΔTV_PET_ was 39.6 ± 52.1%. Baseline SUV_max_ and SUV_mean_ demonstrated a poor correlation with lesion-based response (*p* = 0.706 and *p* = 0.071, respectively). In contrast, SUV_maxT/L_ and SUV_meanT/L_ were significantly correlated with lesion-based response (SUV_meanT/L_: *p* = 0.011, r = 0.412; SUV_maxT/L_: *p* = 0.004, *r* = 0.434). Among patient characteristics—including primary tumor origin, baseline tumor volume, and metastatic sites—only pancreatic origin was significantly associated with functional tumor volume reduction (ΔTV_PET_%: 56.8 ± 39.8 in pancreatic vs. 28.4 ± 50.1 in non-pancreatic NET; *p* = 0.020).

**Conclusion:**

The lesion-based molecular response to PRRT correlates with pretreatment somatostatin receptor PET uptake, particularly when expressed as tumor-to-liver SUV ratios (SUV_maxT/L_ and SUV_meanT/L_).

## Introduction

Neuroendocrine tumors (NETs) are rare neoplasms that originate from endocrine or neuroendocrine cells (1). In the United States, the incidence rate was 8.19 cases per 100,000 individuals in 2018 (2). The gastroenteropancreatic (GEP) region is the most common primary site, although NETs can arise in various other locations. Histological grading was traditionally determined using markers such as mitotic count and the Ki-67 index (3); however, it is now primarily based on cell morphology (4). To date, surgical resection remains the first-line treatment for localized disease. The expression of somatostatin receptors (SSTRs) in NETs has been effectively utilized for both diagnostic and therapeutic purposes, resulting in significant tumor load reduction and a favorable safety profile (5–8). More specifically, peptide receptor radionuclide therapy (PRRT) using somatostatin receptor analogs, such as [^177^Lu]Lu-octreotate (^177^Lu-PRRT) or [^90^Y]Y-octreotate (^90^Y-PRRT), has proven to be an effective systemic treatment for unresectable or metastatic neuroendocrine tumors (NETs), yielding remarkable clinical outcomes with low overall toxicity (9–17). The therapeutic benefit of PRRT was demonstrated in the NETTER-1 trial (18), which led to the FDA approval of [^177^Lu]Lu-DOTATATE in 2018. As an integral part of NET diagnostics, somatostatin receptor scintigraphy with [^111^In]In-DTPA-octreotide and, more recently, positron emission tomography (PET) using ^68^Ga-labeled somatostatin analogs, such as [^68^Ga]Ga-DOTA-Tyr3-octreotide (DOTATOC), has been established as a superior imaging modality (19–22). In addition to diagnosis, staging, and therapy response evaluation, somatostatin receptor imaging is also crucial for patient selection, ensuring that only those with adequate SSTR expression receive PRRT. However, the heterogeneous SSTR across various tumor lesions results in significant intra-individual variability in tracer uptake on pre-therapeutic ^68^Ga-DOTATOC PET/CT scans of SSTR-expressing NET patients undergoing PRRT (23). This study aims to investigate the relationship between lesion-specific baseline SSTR expression and tumor response to [^177^Lu]Lu-octreotate, as measured by tumor volume change following treatment.

## Materials and methods

### Patients’ characteristics and PRRT

This retrospective analysis included a total of 32 patients with histologically confirmed, unresectable, metastatic gastroenteropancreatic neuroendocrine tumors (GEP-NET) who underwent treatment with [^177^Lu]Lu-octreotate (17 men, 15 women; age range: 40–90 years; mean age: 67.8 years; median age: 70 years). Prior to PRRT, patients underwent various pre-treatments, including surgical resection, somatostatin analog (SSA) therapy, targeted molecular therapies (e.g., everolimus, sunitinib), or chemotherapy, depending on tumor burden, progression status, and individual patient characteristics. All patients met the general inclusion criteria for peptide receptor radionuclide therapy (PRRT), including sufficient tumor uptake (i.e., uptake ≥ liver uptake) on baseline [^68^Ga]Ga-DOTATOC-PET/CT (24–26). Within the cohort, 12 patients had pancreatic NET, while 20 patients had non-pancreatic GEP-NET. The study was conducted in accordance with the Declaration of Helsinki and national regulations. Written informed consent was obtained from all participants for the scientific analysis of their data.

PRRT was administered with a mean activity of 7.9 GBq (216 mCi) [^177^Lu]Lu-octreotate per treatment cycle, targeting a total of four cycles at standard intervals of 3 months (10–14 weeks). The ^177^Lu (IDB Holland, Baarle-Nassau, Netherlands) had a specific activity ranging from approximately 100 to 160 GBq/μmol at the time of administration. Peptide labeling was conducted to achieve an apparent specific activity of approximately 54 GBq/μmol, defined as the ratio of activity to the total peptide amount (27, 28). Nephroprotection was provided through standard amino acid co-infusion following the Rotterdam protocol, consisting of lysine (2.5%) and arginine (2.5%) in 1 L of 0.9% NaCl, administered at an infusion rate of 250 mL/h (29, 30).

### Somatostatin receptor PET-imaging and lesion-based response assessment

Baseline [^68^Ga]Ga-DOTATOC PET/CT was performed 2 to 7 days prior to the first PRRT cycle. Long-acting somatostatin analogs were discontinued for at least 4 weeks, while short-acting analogs were paused for at least 1 day before imaging. DOTATOC labeling was conducted using ^68^Ga eluted from an in-house ^68^Ge/^68^Ga generator, following the procedure described by Zhernosekov et al. (31). The PET/CT scans covered the area from the base of the skull to the upper thighs, with five to seven bed positions, and were acquired 30 min after the intravenous injection of 200 MBq [^68^Ga] Ga-DOTATOC. Imaging was performed using a hybrid PET/CT scanner (Biograph 2, Siemens Medical Solutions Inc., Hoffman Estates, Illinois, United States), which consisted of a dual-detector helical CT and a high-resolution PET scanner with a 16.2 cm axial field of view and lutetium oxyorthosilicate (LSO) crystal detectors (6.45 × 6.45 × 25 mm). CT imaging was performed for attenuation correction and anatomical localization, with acquisition parameters set to a tube current of 60 mAs, a tube voltage of 130 kV, a rotation time of 0.8 s, a slice thickness of 5 mm, a slice width of 5 mm, and a table feed of 8 mm per s. To enhance vascular and parenchymal delineation, 140 mL of iodinated contrast material (Ultravist 300; Schering, Berlin, Germany) was administered via an automated injector (XD 5500; Ulrich Medical Systems, Ulm, Germany) with a start delay of 50 s. Following CT image acquisition, PET data were collected for 5 min per bed position (total duration: approximately 35 min). The PET scanner had a coincidence time resolution of 500 ps, a coincidence window of 4.5 ns, and a sensitivity of 5.7 cps/kBq at 400 keV. Attenuation-corrected PET data were reconstructed using a standardized ordered-subset expectation maximization (OSEM) iterative reconstruction algorithm with two iterations, eight subsets, and a 5 mm Gaussian filter.

For each patient, two to three tumor lesions were selected as target lesions, specifically those that were well-demarcated. Irregular regions of interest (ROIs) with a threshold of 50% of the maximum DOTATOC uptake were drawn on the transverse PET slices. The standardized uptake values (SUV), including SUV_mean_ and SUV_max_, were calculated for each lesion using the standard formula that accounts for the measured activity concentration, corrected for body weight and injected activity. To normalize tumor SUV values, normal liver parenchyma was used as the background reference, and the SUV ratios of target lesions to the liver (SUV_meanT/L_ and SUV_maxT/L_) were derived. Functional tumor volume (TV_PET_) was also determined for each lesion using the same threshold. Restaging with [^68^Ga]Ga-DOTATOC PET/CT was performed 3 months after the completion of PRRT, following the same imaging protocol as at baseline. The response of each tumor lesion was assessed based on the percentage change in functional tumor volume (%∆TV_PET_). In the case of ∆TV_PET_, variations are expressed as absolute values, whereas for %∆TV_PET_, variations are presented as percentages.

The CT-based tumor volume (TV_CT_) was manually segmented and measured using Sectra IDS7 PACS (Version 24.2). ΔTV_CT_ was defined as the absolute change in tumor volume between baseline and post-PRRT imaging, while %ΔTV_CT_ represents the relative volume change normalized to baseline volume. In the case of ∆TV_CT_, variations are expressed as absolute values, whereas for %∆TV_CT_, variations are presented as percentages.

Data are presented using descriptive statistics, including median (minimum–maximum), mean ± standard deviation, and count (percentage). Chi-squared tests or Fisher’s exact tests (as appropriate) were used to compare the proportions of patient groups dichotomized based on baseline characteristics. Mann–Whitney *U* tests were applied to compare quantitative tumor parameters (SUV_max_, SUV_mean_, SUV_meanT/L_, and SUV_maxT/L_) across different groups. The association between tumor parameters in baseline [^68^Ga]Ga-DOTATOC PET/CT and the respective response to PRRT (%∆TV_PET_) was assessed using Spearman’s rank correlation analysis. All tests were two-sided, and a *p*-value <0.05 was considered statistically significant. Statistical analyses were conducted using SPSS (version 20.0; SPSS Inc., Chicago, IL, United States) and GraphPad Prism (version 10.2.3).

## Results

### Patient demographics

A total of 121 PRRT cycles with [^177^Lu]Lu-octreotate were administered to 32 patients. The mean age of the cohort was 67.8 years (range 40–90 years, median 70 years). Patients received up to four PRRT cycles, with a mean of 3.8 ± 0.7 cycles. The mean cumulative activity of [^177^Lu]Lu-octreotate was 29.3 ± 0.7 GBq. Treatment response, assessed according to the modified SWOG criteria (32), included partial response (PR) in 12 patients (37.5%), minimal response (MR) in eight patients (25%), stable disease (SD) in eight patients (25%), and progressive disease (PD) in four patients (12.5%). Therefore, we used post-PRRT PET/CT as the gold standard for response assessment, as it provides functional information on tumor activity. The mean progression-free survival (PFS) was 28.6 ± 15 months. No carcinoid crises were observed.

### Tumor parameters

Lesion-based response analysis following PRRT was conducted for 66 lesions. At baseline, the mean SUV_max_ was 28.1 ± 16 (range: 3.0–91.2), SUV_mean_ was 13.3 ± 5.1 (range: 2.4–27.1), SUV_meanT/L_ was 3.6 ± 1.7 (range: 0.96–10.45), and SUV_maxT/L_ was 7.7 ± 5.6 (range: 1.3–30.9). The functional tumor volume at baseline (TV_PET_) was 53.1 ± 12.2 mm^3^. Moreover, 3 months after PRRT completion, the absolute functional tumor volume change (∆TV_PET_) was 25.5 ± 9.3 mm^3^, while the percentage change in functional tumor volume (%∆TV_PET_) was 40.2 ± 49.7%.

**Figure 1:**
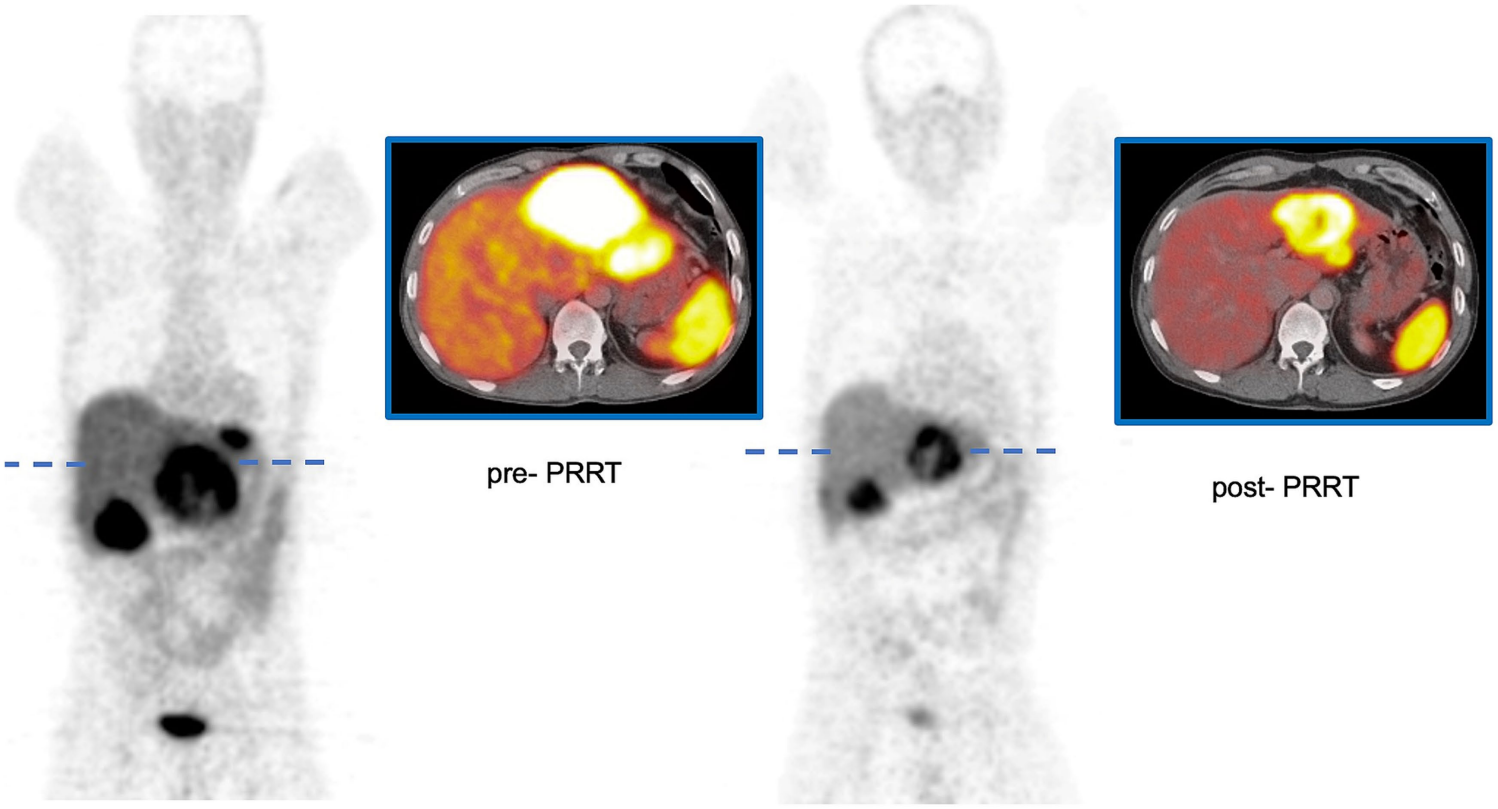
Patient with P-NET before and 3 months after the completion of PRRT using [^177^Lu]Lu-octreotate. ∆TV_PET_ of the large lesion in the left lobe of the liver was 288 mL, with a %∆TV_PET_ of 65%. The SUV_maxT/L_ was 7.0 before PRRT and 5.1 after PRRT.

Some discrepancies between contrast-enhanced CT and [^68^Ga]Ga-DOTATOC PET/CT were observed, particularly regarding detectability and tumor size assessment. Figure 1 illustrates an example of a patient with a pancreatic neuroendocrine tumor (P-NET) before and 3 months after the completion of PRRT. In the lesion-based analysis, neither metastatic site (hepatic vs. extrahepatic, *p* = 0.702) nor baseline lesion volume (*p* = 0.480) significantly influenced lesion response. However, lesions originating from the pancreas showed a significantly greater response compared to non-pancreatic lesions (%∆TV_PET_ 56.8 ± 39.8 vs. 28.4 ± 50.1; *p* = 0.020). Pretreatment SUV-derived values and treatment-induced volumetric changes, stratified by baseline patient characteristics, are summarized in Table 1 (∆TV_CT_, %*∆*TV_CT_) and Table 2 (∆TV_PET_, %*∆*TV_PET_). Since Ki-67 index data were not available for all patients and FDG-PET/CT follow-up data were missing for two patients, 26 and 24 patients, respectively, were analyzed in the tables. No significant difference in CT-derived tumor volume change (∆TV_CT_) was observed based on tumor type (GEP-NET vs. P-NET), overall response (responders vs. non-responders), Ki-67 status, or metastatic location (liver vs. other sites). However, %∆TV_CT_ differed significantly between responders (66.7 ± 39.2) and non-responders (12.7 ± 32.4), while no significant difference was found for other parameters.

**Table 1:** Different pretreatment SUV and volume response parameters according to the patient and tumor characteristics.

	Patients		Lesions		SUV_max_		SUV_mean_		Ratio max/max		Ratio mean/mean		TV_CT 0_ (mL)		∆TV_CT_ (mL)		∆ TV_CT_ (%)	
	*N*	%	*N*	%	Mean ± SD	*p*	Mean ± SD	*p*	Mean ± SD	*p*	Mean ± SD	*p*	Mean ± SD	*p*	Mean ± SD	*p*	Mean ± SD	*p*
Tumor-type																		
GE-NET	19	73	46	73	27.2 ±16.8	0.477	17 ±13	0.814	6.3 ±6.9	**0.012**	5.7 ±9.1	0.099	21 ±43	0.853	4.8 ±18.8	0.161	37.4 ±43.4	0.52
P-NET	7	27	17	27	25.1±7.3		14.8±4.7	0.814	7.7±3.0		5.9±2.6		38.6±107		24.5 ± 69		63.4 ± 45.3	
Ki-67																		
≤2%	9	34.6	22	35	31.2 ±10.5	0.11	18.3 ±6.3	0.0	7.2 ±3.1	0.052	5.5 ±2.6	0.060	14.5 ±21.8	0.841	1.3 ±4.1	0.102	33.1 ±46.4	0.367
>2%	17	65.4	41	65	24.2 ±16.3		15.5 ±12.5	64	6.4 ±7.2		5.9 ±9.5		31.5 ±80.1		14.9 ±48.6		50.5 ±43.7	
Overall response																		
Responder	9	65.4	37	58.7	25.1 ±16.9	**0.020**	16.1 ±12.9	0.1	7.4 ±7.4	0.194	6.8 ±9.9	0.150	28.6 ±81.7	0.240	16.9 ±60.6	0.102	66.7 ±39.2	**<001**
Non responder	17	34.6	26	41.3	28.9 ±11.2		17.1 ±6.9	81	5.6 ±3.1		4.2 ±2.5		21.3 ±34.5		0.4 ±5.6		12.7 ±32.4	
Site (mets)																		
Liver	22	73.4	53	84	24.6 ±10.8	0.250	15 ±6	0.1	5.9 ±4.1	0.460	4.8 ±3.5	0.060	28.2 ±71.7	0.860	10.8 ±43.1	0.965	43.6 ±46.7	0.692
Other	8	26.6	10	16	37.5 ±26.1		24 ±23	40	11 ±12		10.9 ±17.7		11.6 ±9.4		6.5 ±9.1		49.2 ±37.3	
Lesion vol.																		
≤10 mL			40	63.5	24.6 ±10.9	0.287	14.9 ±5.9	0.4	6.1 ±2.9	0.673	4.8 ±2.5	0.596	4.1 ±2.9	**<0.001**	1.98 ±2.9	**0.004**	52.4 ±46.7	0.088
>10 mL			23	36.5	30.3 ±19.7		19.1 ±15.7	63	7.7 ±9.3		7.4 ±12.5		62.9 ±99.9		24.3 ±63.8		30.6 ±39.2	

Bold values indicates statistically significant of *p* <0.05.

**Table 2:** Potential predictors and volume response based on PET (VOI50).

	Patients		Lesions		SUV_max_		SUV_mean_		Ratio max/max		Ratio mean/mean		TV_PET 0_ (mL)		∆TV_PET_ [mL]		∆ TV_PET_ (%)	
	*N*	%	*N*	%	Mean ± SD	*p*	Mean ± SD	*p*	Mean ± SD	*p*	Mean ± SD	*p*	Mean ± SD	*p*	Mean ± SD	*p*	Mean ± SD	*p*
Tumor-type																		
GE-NET	17	70.8	39	70.9	28.7 ±15.7	0.699	13.2 ±4.8	0.770	6.6 ±4.4	0.228	3.1 ±1.24	**0.018**	50.1 ±58.8	0.865	14.1 ±50.6	0.092	27.1 ±54.3	**0.041**
P-NET	7	29.2	16	29.1	33.6 ±18.9	0.699	14.4 ±5.9	0.770	8.2 ±4.9		2 ±1.6		104.8 ±184.4		74.9 ±133.8		70.1 ±29.5	
Ki-67																		
≤2%	9	37.5	23	41.8	25.4 ±12.5	0.353	12.2 ±4.8	0.125	5.9 ±3.6	0.496	3.1 ±1.1	0.61	34.1 ±34.1	0.128	0.84 ±28.8	**0.016**	37.7 ±52.3	0.828
>2%	15	62.5	32	58.2	33.5 ±18.7		14.5 ±5.3		7.4 ±5.2		3.7 ±1.6		88.9 ±140.2		54.1 ±106.5		41.1 ±52.6	
Response																		
Resp.	17	70.8	40	72.7	30.3 ±18.8	0.345	12.8 ±5.3	**0.007**	7.5 ±4.9	0.081	3.6 ±1.5	0.503	71.1 ±128.5	0.639	47.7 ±95.5	**0.003**	59.1 ±35.9	**0.001**
No Resp.	7	29.2	15	27.3	29.7 ±9.67		15.7 ±4.2		5.1 ±2.8		3.1 ±1.3		52.4 ±44.2		10.5 ±32.6		12.3 ±53.7	
Site (mets)																		
Liver	18	58	40	73	27.1 ±12.8	0.177	12.9 ±4.9	0.503	6.1 ±3.5	0.106	3.3 ±1.3	0.106	73 ±123	0.547	30.5 ±94.7	0.280	33.9 ±57.8	0.138
Other	13	42	15	27	38.2 ±22.9		15.2 ±5.6		8.8 ±6.4		3.9 ±1.7		47 ±75		35.4 ±64.3		55.1 ±28.1	
Lesion vol.																		
≤10 mL			15	73	18.5 ±7.4	**0.001**	9.7 ±4.4	**0.001**	4.7 ±1.4	**0.012**	2.7 ±0.6	**0.045**	7.2 ±2.2	**<0.001**	4.3 ±3.7	**<0.001**	50.5 ±58.9	0.138
>10 mL			40	27	34.5 ±17.2		14.9 ±4.6		7.6 ±5.1		3.7 ±1.5		88.1 ±124.5		42.2 ±100.3		53.6 ±49.4	

Bold values indicates statistically significant of *p* <0.05.

Similarly, the change in PET-derived tumor volume (∆TV_PET_) did not show significant differences based on metastatic location or tumor type. Patients with a high Ki-67 index (>2%) exhibited a significantly greater ∆TV_PET_ (54.1 ± 106.5 mL) compared to those with low Ki-67 (<2%) (0.84 ± 28.8 mL). Furthermore, ∆TV_PET_ was significantly higher in overall responders (47.7 ± 95.5 mL vs. 10.5 ± 32.6 mL). Regarding tumor type, %∆TV_PET_ was significantly greater in P-NETs (70.1 ± 29.5) than in GEP-NETs (27.1 ± 54.3). Additionally, overall responders exhibited a significantly higher %∆TV_PET_ (59.1 ± 35.9 vs. 12.3 ± 53.7). However, no significant differences in %∆TV_PET_ were observed based on Ki-67 status or metastatic location.

**Figure 2:**
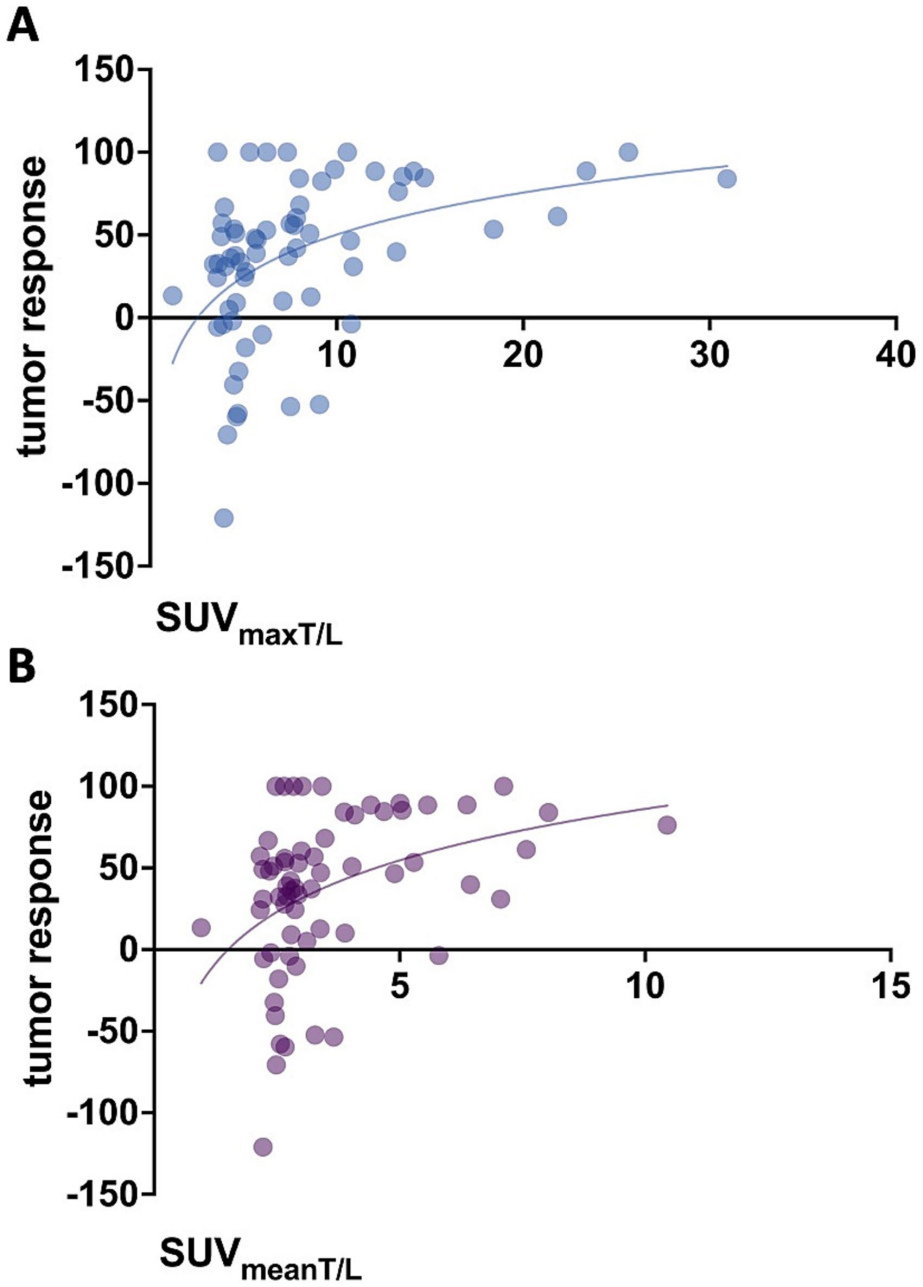
Association between baseline tumor-to-background ratios and tumor response (%∆TV_PET_). **(A)** SUV_maxT/L_ (*p* = 0.004, *r* = 0.435) and **(B)** SUV_meanT/L_ (*p* = 0.011, *r* = 0.381).

**Figure 3:**
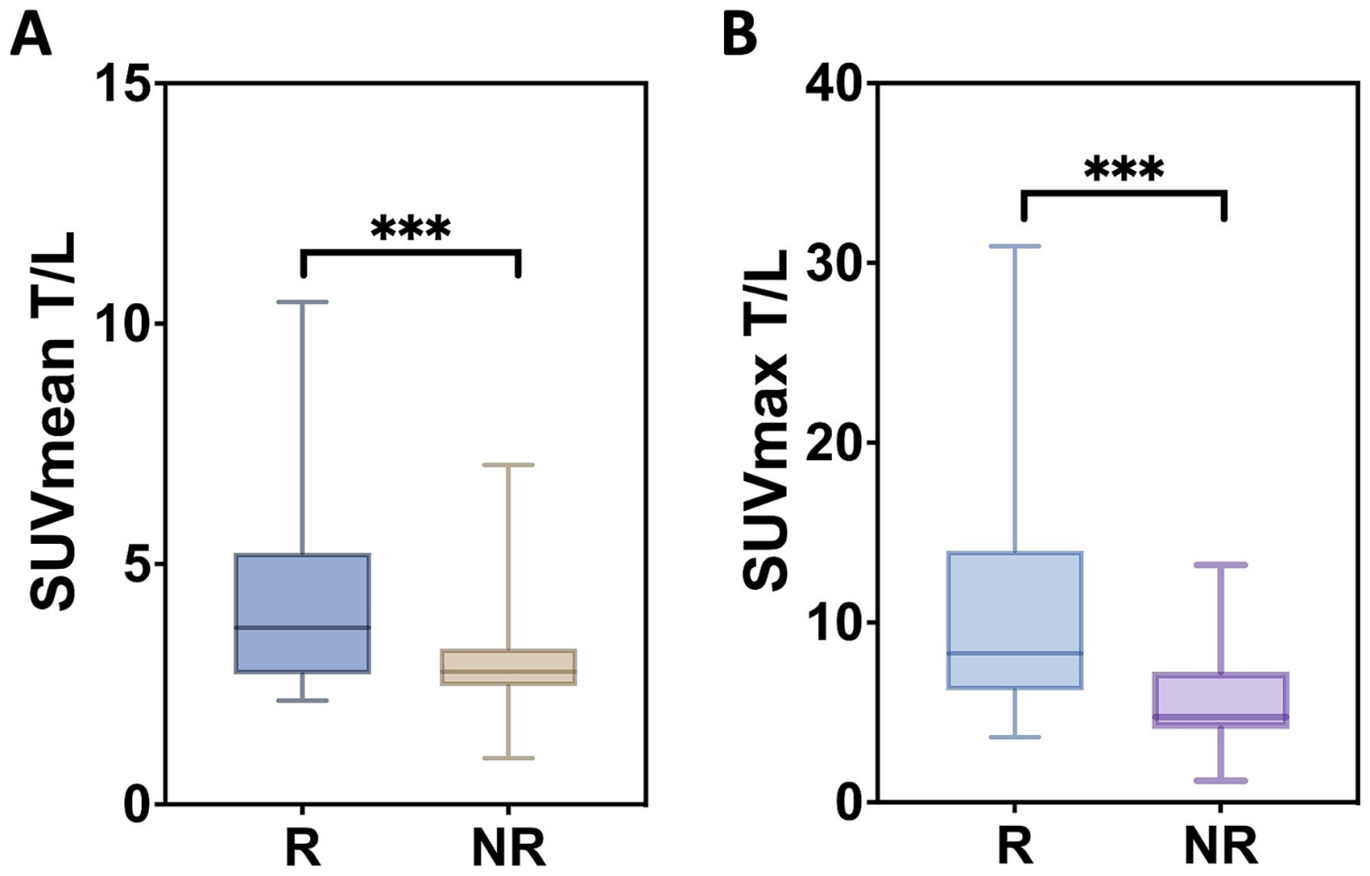
Lesion-based measures. The distribution of SUV_mean T/L_ **(A)** and SUV_max T/L_ **(B)** is shown. Box plots depict the median as well as the upper and lower quartiles of each distribution. R, response; NR, no response. (***) The difference was significant with *p* = 0.027 and *p* = 0.001, respectively.

Baseline SUV_mean_ (*p* = 0.071) and SUV_max_ (*p* = 0.706) of tumor lesions showed no significant association with lesion-based response. In contrast, higher tumor-to-background ratios at baseline, specifically SUV_meanT/L_ (*p* = 0.011, *r* = 0.381) and SUV_maxT/L_ (*p* = 0.004, *r* = 0.435), were associated with more pronounced changes in functional tumor volume, as illustrated in Figure 2. For marker lesions, higher SUV_meanT/L_ and SUV_maxT/L_ were predictive of therapy response, defined as a >50% decrease in %∆TV_PET_ (*p* = 0.0027 and *p* = 0.0001, respectively), as shown in Figure 3; however, a considerable overlap was observed.

Given the higher likelihood of complete remission in smaller lesions, the association between SUV-derived values and lesion-based response was further evaluated for lesions >10 mL (*n* = 44). In this subgroup, the correlation between pretreatment SUV_maxT/L_ (*p* < 0.001) and SUV_meanT/L_ (*p* < 0.001) with functional tumor volume response remained statistically significant (data not shown).

## Discussion

PRRT is a well-established treatment option for advanced NET following the failure of SSA therapy. A primary prerequisite for PRRT is the overexpression of somatostatin receptors (SSTR) on neuroendocrine tumor cells, enabling sufficient tracer uptake to generate high-contrast imaging between tumor lesions and healthy organs. Currently, [^68^Ga]Ga-DOTATOC PET/CT is the preferred modality for assessing SSTR expression. In this study, SSTR expression in tumor lesions was evaluated using [^68^Ga]Ga-DOTATOC PET/CT, with receptor density quantified by tumor-to-liver SUV ratios (SUV_maxT/L_ and SUV_meanT/L_). The findings demonstrate a significant association between receptor density in SSTR-expressing tumor lesions and the response to PRRT based on lesions. This association remained statistically significant even after excluding lesions <10 mL.

The predictive value of pre-therapeutic SUV parameters derived from [^68^Ga]Ga-DOTATOC PET/CT for assessing response to PRRT remains controversial, as previous studies have reported conflicting results. While some studies have identified SUV_max_ as a predictor of treatment response (33–38), others have found no significant association (39–44). For instance, Gabriel et al. (40) reported that baseline SUV_max_ values of the most prominent lesion were comparable between morphologically assessed responders and non-responders to PRRT. Conversely, other studies suggested that higher SUV_max_ values were predictive of treatment response and longer time to progression (33, 45). However, lesion-based analyses were conducted in only three of these studies (34, 37, 44). The lesion-based analysis in our study demonstrated no significant association between baseline SUV_mean_ or SUV_max_ of tumor lesions and lesion-based response. The superior predictive value of baseline tumor-to-liver SUV ratios, compared to tumor SUV_mean_ and SUV_max_, further highlights the limitations of SUV parameters as direct surrogates for somatostatin receptor density and underscores the importance of normalizing these values to background activity.

In addition to [^68^Ga]Ga-DOTATOC PET/CT, [^18^F]-FDG PET/CT has also been shown to play a role in predicting tumor response, disease progression, and survival in patients undergoing PRRT for advanced NET. High [^18^F]-FDG SUV_max_ has been associated with poor clinical outcomes and increased disease progression (34, 46, 47). Based on these findings, [^18^F]-FDG PET/CT may serve as a valuable additional tool for therapeutic decision-making. Another important predictor of PRRT response is the proliferation status of the tumor, as quantified by the Ki-67 index. In our study, Ki-67 >2% was significantly associated with a higher ∆TV_PET_ compared to Ki-67 ≤2%. The proliferation rate is a well-established determinant of survival and a recognized prognostic factor in NETs (48). There is substantial evidence supporting its predictive value for progression-free survival and treatment outcomes following PRRT (11, 48–51), although NETs within the higher G2 range may exhibit treatment responses similar to those with a low Ki-67 index. A recent study introduced an algorithm that incorporates circulating NET transcripts and the Ki-67 index, which correlates with treatment response and effectively predicts PRRT efficacy (52). Another important factor in PRRT treatment decision-making is the quantification of liver tumor burden. Several studies have indicated that patients with a low liver tumor burden achieve significantly longer disease-free survival following PRRT compared to those with a high liver tumor burden (11, 13, 53). In our study, the mean %∆TV_PET_ in liver metastases was lower than in metastases at other locations; however, no significant difference was observed in ∆TV_PET_ or %∆TV_PET_. Further differentiation between metastatic sites may provide additional insights. For example, one study reported that patients with bone metastases had a higher risk of disease progression following PRRT (54).

In this study, the functional tumor volume change (%∆TV_PET_) was chosen as the primary parameter for lesion-based response assessment because evaluating the response of NETs to PRRT using only computed tomography (CT) has shown limited accuracy, particularly in cases of hepatic metastases. Morphological shrinkage is observed in only a minority of patients who demonstrate clear clinical improvement, and anatomical alterations may persist for a prolonged period post-treatment, despite significant local tumoricidal effects (55, 56). When analyzing patient-based characteristics, only tumors of pancreatic origin were significantly associated with greater %∆TV_PET_ volume changes (*p* = 0.041). This finding aligns with previous observations that pancreatic NETs exhibit a more pronounced response to PRRT based on morphological response criteria such as WHO, RECIST, and SWOG (54, 57).

Our study demonstrated a significant correlation between lesion SUV_meanT/L_ and SUV_maxT/L_ and lesion-based response, quantified by %∆TV_PET_. These parameters may serve as valuable tools to support clinical decision-making regarding PRRT eligibility. A lesion-based evaluation may help refine patient selection and treatment planning for PRRT, leading to a more personalized approach. Other factors to consider in this process include [^18^F]-FDG uptake, Ki-67 status, and liver tumor burden. However, further studies are required to identify the optimal patient and tumor characteristics for PRRT selection.

Serological markers were not systematically included in our analysis; however, their potential relevance, particularly chromogranin A levels, as additional indicators of treatment response should be considered. Nonetheless, its suitability as a marker for therapy response under PRRT remains controversial (58). A promising emerging approach for predicting treatment response is radiomics (59). Radiomics involves the extraction and analysis of large-scale quantitative imaging features from medical scans, enabling a more precise prediction of patient outcomes (60). Future research should focus on exploring the potential of radiomics-based models to enhance treatment stratification and response assessment in PRRT.

This study has several limitations. First, the analysis was retrospective, observational, and conducted at a single center, which may limit generalizability. Second, the sample size was relatively small, with only 32 patients included in the retrospective analysis. We emphasize the exploratory nature of our findings and acknowledge the need for larger, prospective studies to confirm our results. Additionally, the administered activity of [^177^Lu]Lu-octreotate varied among patients, with a mean cumulative activity of 29.3 ± 0.7 GBq across 3.8 ± 0.7 cycles.

In conclusion, the lesion-based molecular response to PRRT is significantly associated with pretreatment somatostatin receptor uptake, quantified by tumor-to-liver SUV ratios in [^68^Ga]Ga-DOTATOC PET.

## Data Availability

The raw data supporting the conclusions of this article will be made available by the authors, without undue reservation.
